# Frequency of Oral Impacts on Daily Performances and Dental Pain Among Indigenous Adolescents of Himalayas (Leh, Ladakh): A Cross-Sectional Study

**DOI:** 10.3290/j.ohpd.b965717

**Published:** 2021-02-19

**Authors:** Kuldeep Singh Shekhawat, Srinivasan Raj Samuel, Arunima Chauhan

**Affiliations:** a Associate Professor, Department of Public Health Dentistry, Century International Institute of Dental Sciences, Kerala, India. Concept, design, literature search, data acquisition, data analysis, statistical analysis, manuscript preparation, contributed to discussion.; b Associate Professor, Department of Public Health Dentistry, Saveetha Dental College, SIMATS, Chennai, Tamil Nadu, India. Data acquisition, literature search, contributed to discussion.; c Professor, Faculty of Dentistry, Department of Oral Biology, Melaka Manipal Medical College (Manipal Campus), Manipal Academy of Higher Education, Manipal, Karnataka, India. Concept, manuscript preparation, editing, contributed to discussion.

**Keywords:** adolescent, child, quality of life, school health services, dental care, dental pain

## Abstract

**Purpose:**

Psychosocial impacts on quality of life among adolescents with access to affordable dental care is not well documented. In addition, dental pain is accelerating towards a public health problem that needs immediate attention. The objective was to determine impacts on quality of life using the Oral Impacts on Daily Performances (OIDP) frequency scale and to determine prevalence of dental pain with its impact.

**Methods:**

A total of 288 students (mean age 15.72 ± 1.5) completed the survey instrument (sociodemographic variables, consumption of chocolates/candies, perceived need for dental care, history of dental pain in last 6 months and OIDP frequency scale) designed to measure subjective oral health indicators. Mean OIDP simple count scores were analysed using logistic regression and additive (ADD) scores for dental pain were compared using student’s t test.

**Results:**

The response rate was 96%. About 44.4% reported impacts affecting daily performances. About 11.4% consumed tobacco and 92.7% consumed forms of refined sugars. About 39% perceived a need for dental care and 32.3% experienced dental pain with problem in eating and cleaning teeth. Those not perceiving a need for dental care were more likely to have an impact (OR: 2.3; CI: 1.2–4.4). Males had higher OIDP ADD scores for dental pain than females (p = 0.015).

**Conclusion:**

Overall impact was less than 50%. Dental pain was reported among students with access to dental care with impacts on eating and cleaning of teeth. Oral health promotion needs to be reinforced by strengthening school community relationship.

Oral health is fundamental to general health enabling an individual to eat, speak and socialise. According to World Health Organization, oral diseases are one of the most common non-communicable^29^ and costly diet–lifestyle-related disease^26,30^ affecting 3.5 billion people worldwide. More importantly, most oral health problems are reversible and preventable during their onset, failing which can result in pain and tooth loss.^29^ Dental pain is the most common symptom prompting patients to seek dental treatment.^9^ These are more frequent, have a negative impact on daily life and are often found to limit the expected roles of school-aged adolescents aged 18 years or younger.^14^

An important constituent of providing dental care is also improvement in the quality of life since most oral diseases (and their consequences) often interfere with, or have impacts on daily performances.^8^ The oral well-being of people and especially school students in terms of feelings about their mouths is ignored when measured traditionally using clinical dental indices which focus on absence or presence of diseases.^4^

Schools provide an effective platform for promoting oral health. Using the structures and systems already in place, a school can be an efficient setting where oral health services can be integrated in their previously adopted and routinely followed school health services.^16^ Another advantage of using schools as a venue for oral health promotion/prevention activities and/or comprehensive treatment, is for the reason that a sizeable number of students of different age groups are available for a fixed duration of time. This is more beneficial especially for those schools situated in remote and rural parts of any country.

The objective of the study was to determine the frequency of oral impacts on daily performances experienced by indigenous adolescents using the Oral Impacts on Daily Performances (OIDP) frequency scale. The secondary objective was to determine the prevalence of dental pain and its impact.

## Materials and Methods

### Area Profile

District Leh is situated roughly between 32- and 36-degree north latitude and 75–80-degree east longitude and altitude ranging from 2300 meters to 5000 meters above sea level. It has a total area of 45,100 km^2^. Topographically, the whole of the district is mountainous with three parallel ranges of the Himalayas, the Zanskar, the Ladakh and the Karakoram. During winter season the highway road (Zojila Pass) remains closed because of heavy snowfall, with temperatures dropping up to minus 30^o^C. As a result the region remains cut off for 6 months from rest of the world. It has a population of 133,487 with a literacy rate of 77.2%. About 65.7% of the population reside in a rural area.^10^ There are two private dental practitioners and three dentists working at the government hospital, indicating a less than optimal oral health care services with poor public transport.^27^ The district of Leh has been declared as having a tribal population.^11^

### Study Setting

The present cross-sectional questionnaire study was conducted in one conveniently selected private school in the town of Leh (Ladakh). The school was selected since it fulfils the criteria of being an oral-health-promoting school to a large extent. The school has a total strength of 2014 students, with over 60 classrooms. All the students are compulsorily screened for oral diseases. Those requiring treatment are then referred to the clinics where a range of oral diseases (non-life threatening yet having the capability to have an impact of quality of life) are treated free of charge.

### Sample Population

The present study was conducted in a school that is partly residential with hostel facilities for students from other rural areas of Leh (Ladakh). The institutional permission was obtained from the management of the school before involving students. The procedures followed were in accordance with the World Medical Association Helsinki Declaration of 1975, as revised in 2000. Group consent was obtained from the headmaster of the school. The inclusion criteria for the present study were students above the age of 13 years and those present on the day of the study. The sample size for the present study was calculated with a precision of 1.96 at 95% confidence interval, permissible error of 5% and prevalence rate obtained from previous study.^27^ The total sample size was estimated to be 272. Adjusting non-response rate at 10%, the final sample size was 299.2 (300, round figure).

### Study Participants

Students from class VII to XII were the study population. Each grade had three separate sections and each section have an average of about 50 students. This implied we had to select 50 participants from each class/grade. Informed assent was obtained from all the participants before including them in the present study. The study participants were systematically selected from each class, irrespective of their sections. Prior to the study the head teachers of each class were explained about the study and they in turn explained the nature of the study to their students in the presence of investigators. Collection of data was done during school hours (free classes) so as not to disturb their academic curriculum.

### Survey Instrument

A self-administered closed-ended questionnaire was designed in English, the medium/language of instructions in the school. Therefore, the translation and back-translation of the questionnaire was not required. Apart from sociodemographic characteristics, study participants were also asked, ‘whether they consumed refined sugar (chocolate and candies) during and/or after the schools and the frequency of the same’. In addition, any history of dental pain in the last 6 months and their perceived need for dental care was also elicited. The response was dichotomised as ‘yes’ or ‘no’. The OIDP inventory formed the last part of the questionnaire. For analysis, age was categorised as those below 15 years (<15 years) and at or above 15 years (≥15 years).

### Oral Impact on Daily Performances (OIDP)

The OIDP scale^2^ assesses impacts that affect individuals’ daily life. It is based on an explicit conceptual framework, the World Health Organization’s International Classification of Impairments, Disabilities and Handicaps, ICIDH,^6^ which has been amended for dentistry by Locker.^18^ The ICDIH has the key concepts of impairments, functional limitation, pain and discomfort, and disability and handicap. The OIDP concentrates only on the third level of measurement (pain and discomfort) and is calculated by multiplying frequency and severity scores of daily performances. For the present study, we used the unweighted or abbreviated version of the OIDP frequency scale since applications of weighted scores revealed no statistically significant improvements and sociodental indicators were reported to be satisfactory with the unweighted scores.^1,3^

Oral impact of daily performance was obtained by adding scores for eight frequency items, with the following question: ‘During the past 6 months how often have problems with your mouth and teeth caused you any difficulties with (1) eating, (2) speaking and pronouncing clearly, (3) cleaning teeth, (4) sleeping and relaxing, (5) smiling without embarrassment, (6) maintaining emotional state, (7) enjoying contact with other people, and (8) carrying out major school work’. The scale used was in the range: (0) ‘never affected’; (1) ‘less than once a month’; (2) ‘once or twice a month’; (3) ‘once or twice and a week’; (4) ‘3–4 times a week’; (5) ‘every or nearly every day’. For analysis, dummy variables were constructed yielding the categories 0 = ‘never affected’ (including the original category 0) and 1 = ‘affected less than once a month or more often’ (including the original categories 1–5). Simple count scores (SC) were created by adding the eight dummy variables. Additive scores (ADD) were created by adding the eight OIDP items as assessed originally. Finally the OIDP SC frequency scores were dichotomised, yielding the categories (0) ‘no daily performance affected’ and (1) ‘at least one daily performance affected’.

Even though OIDP measures impact due to problems of mouth and teeth, we also utilised the same OIDP inventory for those study subjects who experienced only dental pain. The data was extracted and prevalence estimates of impacts experienced due to dental pain was assessed. The procedure for obtaining scores was same as just mentioned.

Data was analysed using Statistical Package for Social Sciences (SPSS version 15.0, Chicago, IL, USA). Cronbach’s alpha was used for internal consistency reliability. Descriptive analyses were done for frequency distribution. Multivariate analyses with OIDP and impacts as outcome variables were conducted using multiple logistic regression analyses and 95% confidence intervals (CI). Student’s t test was used to compare mean additive score of OIDP (for dental pain) with respect to gender, place of residence and perceived need for dental care.

## Results

The response rate was 96% (288/300) with a mean age of 15.72 ± 1.5. Females were more in proportion than males and about 57% were staying with their parents. Tobacco consumption was observed among 11.8% of students (34/288) and about 92% (267/288) reportedly bought sugar candies/chocolates from shops located outside the school campus. About 12% (37/267) consumed sugar candies/chocolates every day. A total of 39.5% (114/288) of study participants reported a perceived need for dental care (Table 1).

**Table 1:** Distribution of demographic details of study participants

Variable		Mean, SD
**Mean age**		15.72 ± 1.5
		% (N)
**Gender**	Males Females	45.1 (130) 54.8 (158)
**Residence**	At home Hostel	57.6 (166) 42.3 (122)
**Consumption of sugars**	Never Yes	7.29 (21) 92.7 (267)
	Everyday 3 or 4 times/week 1 or 2 times/week 1 or 2 times/month < once month	13.8 (37) 8.6 (23) 22.4 (60) 41.1 (110) 13.8 (37)
**Perceived need for Dental treatment**	Yes No	60.4 (174) 39.4 (114)
**Dental Pain**	Yes No	32.2 (93) 67.7 (195)
**Tobacco consumption**	No Yes	88.1 (255) 11.4 (33)
	Smoking Smokeless	60.6 (20) 39.3 (13)

% = Proportion; N = Frequency; SD = Standard deviation.

### Prevalence of Oral Impact on Daily Performances

#### Overall impact on daily performances among study participants

About 44.4% (128/288) of study participants experienced at least one impact on their daily life. A total of 29.1% and 26.7% of participating students confirmed difficulties with eating and cleaning their teeth. The remaining impacts on sleeping, studying, speaking, social contacts, emotional imbalance and smiling were relatively low (Table 2). The mean OIDP ADD and OIDP SC scores were 3.7 ± 3.6 and 2.06 ± 1.2, respectively.

**Table 2:** Prevalence of impacts among study participants assessed using OIDP (overall impact and impacts due to dental pain)

	Overall impact^a^ (N – 128)		Impact due to dental pain^b^ (N – 93)	
		Mean (1–5)		Mean (1–5)
Eating Speaking Cleaning teeth Smiling Sleeping Emotional imbalance Studying Social contact Total OIDP SC score	29.1 (84) 7.29 (21) 26.7 (77) 3.47 (10) 9.3 (27) 4.1 (12) 8.3 (24) 4.5 (13) 44.4 (128)	0.9 (0.83) 0.24 (0.61) 0.92 (0.95) 0.17 (0.64) 0.31 (0.69) 0.12 (0.41) 0.25 (0.6) 0.10 (0.38) 2.06 (1.2)	21.1 (61) 3.4 (10) 17 (49) 2 (6) 6.2 (18) 2.4 (7) 5.2 (15) 3.1 (9) 32.2 (93)	0.93 (0.85) 0.17 (0.56) 0.78 (0.9) 0.15 (0.62) 0.29 (0.68) 0.10 (0.4) 0.21 (0.54) 0.09 (0.29) 1.88 (1.2)
Total OIDP ADD score		3.7 (3.6)		2.75 (2.5)

Cronbach’s Alpha: 0.74 for OIDP frequency scale

Using multiple logistic regression with impacts reported as outcome variables, it was found that those with no perception for dental care were more likely to have an impact (OR –2.3, CI: 1.2–4.4) compared to their counterparts who perceived a need for dental care (Table 3). Among those reporting at least one impact (44.4%, 128/288) (OIDP SC >1, affected less than once a month or more), females reported more impacts than males and about 53% (69/128) perceived a need for dental care. About 40% (52/128), 31.2% (40/128), and 17.9% (23/128) reported as having had only one impact, two impacts, and three impacts on their daily life performances, respectively (Table 4).

**Table 3:** Odds ratio (OR) and 95% confidence interval for participants OIDP scores (0 = no impacts; OIDP > 0 = 1) by age group, gender, place of residence and perceived need for dental care

	Adjusted OR (95% CI)	p value
**Age group**		
<15 years ≥ 15 years	I 0.57 (0.3–1.05)	0.07
**Gender**		
Male Female	I 0.9 (0.5–1.75)	0.87
**Place of residence**		
Home At hostel	I 0.8 (0.4–1.5)	0.65
**Perceived need for dental care**		
Yes No	I 2.3 (1.2–4.4)	0.001

**Table 4:** Distribution of study participants according to gender, residence, and frequency of impacts experienced (total N = 128)

Variables	% (N)
**Gender**	
Males Females	39 (50) 61 (78)
**Residence**	
At home Hostel	54.6 (70) 45.3 (58)
**Consumption of chocolates/candies outside school campus**	
Yes No	91.4 (117) 8.6 (11)
**Perceived need for dental care**	
Yes No	53.9 (69) 46.1 (59)
**Number of impacts**	
Only 1 impact 2 impact 3 impact 4 or more impacts	40.6 (52) 31.2 (40) 17.9 (23) 9.8 (13)

% = Proportion; N = Frequency.

#### Impact due to dental pain reported by study participants

A total of 32.3% (93/288) of study participants reported dental pain in preceding 6 months. The OIDP ADD scores and OIDP SC scores were 2.75 ± 2.5 and 1.88 ± 1.2, respectively. A total of 21.2% and 17% reported difficulty in eating and cleaning their teeth. The least reported impact due to dental pain was difficulty in smiling (Table 2). Males had statistically significantly higher OIDP ADD scores than females (p = 0.015). There was no statistically significant difference between the mean OIDP ADD scores with respect to place of residence (p = 0.78) and perceived need for oral care (p = 0.768) (Table 5).

**Table 5:** Mean OIDP ADD scores of study participants with impacts due to dental pain

		N	Mean ADD	t	.sig
Gender	Males Females	32 61	3.62 ± 3.4 2.3 ± 1.7	2.46	p = 0.015^*^
Residence	At home Hostel	51 42	2.82 ± 2.8 2.6 ± 2.1	0.295	p = 0.78
Perceived need for dental treatment	Yes No	51 42	2.6 ± 2.1 2.8 ± 2.9	0.277	p = 0.76

*Statistically significant; level of significance at p <0.05; ADD – additive scores.

## Discussion

The participants of the study were familiar with English as a language and thus the need for translation and back-translation of OIDP frequency scale was avoided, thereby eliminating the vigorous procedures required for cross-cultural adaptations of sociodental indicators of local population. The school provides hostel facilities for their students, therefore the present study also had students from different geographical regions of Leh (Ladakh).

A health-promoting school provides an environment that supports and encourages healthy lifestyles.^17^ The school promotes the policy of ‘sugar-free campus’ and therefore any form of refined sugar is not sold over the counter within the school campus. Surprisingly, it was found that 92.7% of the study participant reported consumption of candies/chocolates from shops located immediately outside the school campus. It is rather a limitation that calls for more coordinated efforts towards oral health education and effective preventive programmes in collaboration with public health authorities.

Majority of students perceived a need for dental care indicating a likelihood of experiencing an impact among those who did not. This is a good indicator since perceptions of need for dental care mediates seeking dental care, overcoming one of the barriers in utilisation of oral health care services. It can be assumed that those who perceived a need for dental care, regularly self-evaluate their oral health status. However, it has to be remembered that the school has a dental unit and perhaps this positive indicator could be the result of either routine dental check-up and/or a pending appointment with the resident dentist. Another important finding was the consumption of tobacco among school students. The prevalence among the present study participants was 11.8%, which was more than the findings from Global Adult Tobacco Survey conducted in 2016–17 for adolescents aged 15 years (3.4% for smokers and 10.8% for smokeless tobacco)^13^ and 5.9%,^7^ 5.5%,^22^ and 6.8%, respectively,^25^ reported in literature by various authors. Surprisingly, the youngest study participant to consume both smoking and smokeless form of tobacco in the present study was aged 13 years.

The present study revealed 44.4% of participants experienced an oral impact that affected their daily life in the past 6 months. The impact prevalence rates ranged from 3.4% to 29.1%. The prevalence of experiencing at least one oral impact in the present study was higher when compared to results reported in literature,^21,19^ very similar to a study conducted in India^28^ and lower when compared to a study conducted in Uganda.^5^ The results in the preceding studies ranged from 28.6% to 62%, although difficulty in eating food and cleaning teeth were the impacts most frequently reported.^5,21,28^ Cleaning their teeth was difficult among present study participants when compared to a study conducted in an Indian setting.^28^

In spite of available oral health services that was easily accessible and available free of cost, 32.2% of the study participants experienced dental pain in the preceding 6 months. The prevalence of dental pain reported in literature varies since authors have considered different age groups within adolescence. The reported prevalence of dental pain was almost similar to studies conducted in India, Tanzania and Pakistan where the prevalence reported were 35%, 36.4%, and 30.4%, respectively.^15,19,23^ Nevertheless, the findings was higher than another study conducted among Indian and Brazilian adolescents where a prevalence of 15.6% and 17.5% was reported.^12,24^ We hypothesise that students might not report any incidence of dental pain immediately, perhaps expecting the dentist to elicit the same during their routine dental check-up.

Dental pain in the present study also had an impact on daily activities of adolescents. The impact prevalence rates ranged from 2% to 21.1%. Difficulty in eating and cleaning teeth was the most common impact experienced by adolescents. This indicated that dental pain had more serious consequences on functional than for social and psychological performances. Difficulty eating food was also consistent with results of a study previously conducted among indigenous adolescents of Leh.^27^ In the present study we observed that among those experiencing dental pain males had statistically significantly higher OIDP ADD scores than females and no differences were observed with respect to place of residence and felt need for dental treatment.

In the present study, participants were expected to recall their experience of dental pain for the preceding 6 months, while some studies had a recall time frame of 1 month. The 6-month time was used in the present study, since a period for up to 12 months does not affect the prevalence estimates when it comes to serious experiences.^20^

It is recommended that there is a need to determine the relation between reported dental pain and perceived need for dental care. In the present study, we observed not everybody who experienced dental pain reported need for dental treatment. This indicates a social gradient in impairment coping- or impairment reducing behaviours, suggesting that these adolescents from the Himalayan region possess better ability to cope with functional impairments. This can be attributed to the fact that the present study was conducted in the month of May 2017. Therefore any experience of dental pain in the preceding 6 months would mean a time frame where the temperature drops below ‘zero’ degrees and the dental unit is closed due to severe winters. That is the commutation impairing time of the year in Leh.

A limitation of this study is its cross-sectional nature and hence further studies are required to better understand and interpret impacts arising due to problems in oral cavity. Another limitation is the generalizability of the results. Since the study was conducted in one single school with more than 2000 students, results cannot be extrapolated to other schools. Most of the studies conducted have utilised a child OIDP inventory that uses a relatively short recall time period of 3 months as compared to OIDP that uses a recall period of 6 months. This can lead to a slight underestimation of prevalence rates in the present study.

## Conclusion

It can be concluded that adolescents with access to dental care in an otherwise geographical area deprived of dental services reported impacts affecting their daily performances. Dental pain was prevalent with functional impairments of difficulty eating and cleaning their teeth. Impacts were observed among those not perceiving a need for dental care.
